# Repression of Smad4 by MicroRNA-1285 moderates TGF-β-induced epithelial–mesenchymal transition in proliferative vitreoretinopathy

**DOI:** 10.1371/journal.pone.0254873

**Published:** 2021-08-12

**Authors:** Shu-I Pao, Le-Tien Lin, Yi-Hao Chen, Ching-Long Chen, Jiann-Torng Chen

**Affiliations:** 1 Department of Ophthalmology, Tri-Service General Hospital, National Defense Medical Center, Taipei, Taiwan, Republic of China; 2 Department of Ophthalmology, Tri-Service General Hospital Songshan Branch, National Defense Medical Center, Taipei, Taiwan, Republic of China; 3 Graduate Institute of Medical Sciences, National Defense Medical Center, Taipei, Taiwan, Republic of China; National Institutes of Health, UNITED STATES

## Abstract

The purpose of this study was to assess whether microRNA (miR)-1285 can suppress the epithelial–mesenchymal transition (EMT) in retinal pigment epithelial cells. Expression of miR-1285 was evaluated using quantitative real-time polymerase chain reaction (RT-qPCR). The features of EMT were assessed using Western blotting, immunocytochemical staining, scratch wound healing tests, modified Boyden chamber assay, and collagen gel contraction assay. A rabbit model of proliferative vitreoretinopathy (PVR) was used for in vivo testing, which involved the induction of PVR by injection of transfected ARPE cells into the vitreous chamber. Luciferase reporter assay was performed to identify the putative target of miR-1285. The expression of miR-1285 was downregulated in ARPE-19 cells treated with transforming growth factor (TGF)-β. Overexpression of miR-1285 led to upregulation of zonula occludens-1, downregulation of α-smooth muscle actin and vimentin, cell migration and cell contractility—all EMT features—in the TGF-β2-treated ARPE-19 cells. The reporter assay indicated that the 3′ untranslated region of Smad4 was the direct target of miR1285. PVR progression was alleviated in the miR-1285 transfected rabbits. In conclusion, overexpression of miR-1285 attenuates TGF-β2-induced EMT in a rabbit model of PVR, and the effect of miR-1285 in PVR is dependent on Smad4. Further research is warranted to develop a feasible therapeutic approach for the prevention and treatment of PVR.

## Introduction

Proliferative vitreoretinopathy (PVR), a common cause of severe visual loss or blindness, is a clinical syndrome associated with retinal traction and detachment in which cells with proliferative potential multiply and contract on retinal surfaces and in the vitreous chamber [1]. PVR develops through cellular exposure to growth factors and cytokines, and such exposure involves the breakdown of the blood–retinal barrier (BRB) and of cellular contact with the vitreous gel. In recent years, growing evidence has shown that inflammatory mediators in the vitreous or in subretinal fluid, including growth factors and cytokines, are important players in the occurrence and development of PVR [2–5]. The histological and clinical studies have highlighted the chain of events leading to PVR: cellular migration into the vitreous chamber, cell differentiation, myofibroblast proliferation and activation, synthesis of extracellular matrix proteins, and then the contraction of epiretinal membranes (ERMs) [6]. Multiple cell types have been identified in ERMs, and retinal pigment epithelial (RPE) cells have long been implicated as key players in the pathophysiology of PVR. Both clinical and empirical studies have indicated that RPE cells undergo epithelial–mesenchymal transition (EMT) to express a fibroblastic phenotype. Cell–cell adhesion maintained by adherens junctions and tight junctions (TJs) are essential to maintenance of the RPE phenotype. The disruption of these junctional complexes leads to EMT through the activation of signaling pathways such as transforming growth factor (TGF)-β, platelet-derived growth factor, and connective tissue growth factor [7]. Numerous fibrogenic factors or cytokines participate in EMT, and TGF-β is believed to play a central role [8]. Therefore, the effects of TGF-β signaling regulations on EMT regulation warrants further investigation.

MicroRNAs (miRNAs), which are approximately 18 to 24 nucleotides in length, are small molecules of endogenous, noncoding RNA that function as posttranscriptional regulators. MiRNAs contribute to the regulation of approximately 30%–50% of gene encoding proteins through interactions with the 3′ untranslated region (3′-UTR) of targeted genes [9]. Abnormal expression of miRNA has been reported to regulate gene expression and the growth, invasion, and migration of cancer cells [10]. Many miRNAs are evolutionarily conserved, suggesting that they have essential biological functions [11]. Various microRNAs are believed to act on the retina or on RPE cells [12–15]. Since the discovery of miR-1285 through the sequencing of human embryonic stem cells, it has been linked to cancer [16]. Huang et al. demonstrated that miR-1285 inhibited the migration and invasion of pancreatic cancer cells, acting as a tumor suppressor [17]. In addition, Hironaka-Mitsuhashi et al. observed that the overexpression of miR-1285-5p significantly suppressed the proliferation of breast cancer cells [18]. Taken together, this evidence suggests the possible role of miR-1285 in EMT pathogenesis.

Smad4 is a central signaling component of the Smad pathway that transduces signals from TGF-β [19]. During TGF-β ligand stimulation, Smad4 forms a heteromeric complex with receptor-regulated Smad2/3. This paired complex is phosphorylated by an activated TGFβ receptor complex consisting of two type I and two type II transmembrane serine threonine kinase receptors [20]. Deckers et al. reported that the suppression function of small hairpin RNA (shRNA) against Smad4 led to strong inhibition of TGFβ-induced EMT in breast cancer cells [21]. Another study demonstrated that miR-1285 influenced the proliferation and metastasis of non-small-cell lung carcinoma cells through the downregulation of Smad4 [22]. In the present study, the overexpression of miR-1285 inhibited TGF-β/Smad4 signaling, which reduced both the expression of mesenchymal markers and inhibited the EMT of RPE cells. Moreover, miR-1285 regulated fibroblastic transdifferentiation in RPE cells by targeting the 3′-UTR during EMT.

The present study aimed to assess whether microRNA (miR)-1285 suppresses EMT in retinal pigment epithelial cells. We used an ARPE-19 cell line in an EMT model and a rabbit model of PVR to study the alterations of miR-1285 expression in and its effects on PVR. The results suggest that miR-1285 may contribute to the prevention of PVR by inhibiting TGF-β2-induced EMT via suppressing TGF-β/Smad4 signaling. These findings reveal the potential of miR-1285 as a therapeutic approach against PVR in RPE cells.

## Materials and methods

### Human RPE cells and treatment

A human RPE cell line, ARPE-19, was obtained from the American Type Culture Collection (Manassas, VA, USA). The ARPE-19 cells were cultured in a 1:1 mixture of Dulbecco’s modified Eagle’s medium (DMEM) and Nutrient Mixture F-12 Ham (Invitrogen-Gibco, Grand Island, NY, USA) with 4 mM L-glutamine supplemented with 10% fetal bovine serum (FBS; Invitrogen-Gibco), 100 U/mL penicillin, and 100 μg/mL streptomycin (Sigma-Aldrich, St Louis, MO, USA). The cells were maintained at 37°C in a humidified environment with 5% CO_2_, with media exchange performed twice a week.

The miRNA mimics [miR-1285 and negative control (NC)] and inhibitors (miR-1285 and NC) were purchased from Life Technologies (Carlsbad, CA, USA). ARPE-19 cells were transfected with miRNA mimics or miRNA inhibitors for 24 hours, followed by treatment with or without 10 ng/mL human recombinant TGF-β2 (PeproTech, New York, NY, USA) for an additional 24 hours. The transfection of either siRNA or miRNA (mimic/inhibitor) was accomplished using Lipofectamine 2000 (Thermo Fisher Scientific), according to the manufacturer’s instructions.

### Quantitative analysis of relative miRNA expression

After removal of the culture medium and three rinses with phosphate-buffered saline (PBS), total RNA was extracted from the cells using TRIzol reagent (Life Technologies). Next, 1μg of total RNA aliquot was reverse transcribed using the TaqMan MicroRNA reverse transcription kit. Quantitative real-time PCR (RT-qPCR) was performed using Real MOD Probe SF 2X qPCR mix and the Step One Plus PCR System (Applied Biosystems, Foster City, CA, USA). The miR-1285 expression level was normalized to that of the control (U6 small nuclear RNA) or miR-361 using the 2-ΔΔCT method. The expression level of miR-1285 in the presence or absence of 10ng/mL TGF-β2 in the RPE cells was also evaluated.

### Western blotting

After the experimental treatments, the cells were lysed and sonicated in lysis buffer (50 mMTris-HCl [pH 7.5], 2% sodium dodecyl sulfate, and 1 mM phenylmethylsulfonyl fluoride). The protein content was quantified using the Pierce BCA protein assay kit (Rockford, IL, USA). Specifically, 20 μg of protein in each sample was loaded and subjected to sodium dodecyl sulfate–polyacrylamide gel electrophoresis and then transferred to a polyvinylidene fluoride membrane (Millipore, Billerica, MA, USA). After blocking with 5% skimmed milk dissolved in a phosphate buffer solution with Tween20 at room temperature for 1hour, the membranes were directly probed with primary antibodies againstα-smooth muscle actin (α-SMA; Sigma-Aldrich, St. Louis, MO, USA), anti-zonula occludens (ZO)-1 (Zymed Laboratories, South San Francisco, CA, USA), phosphorylated (p)-Smad2/3 (Cell Signaling), Smad4(1:1000 dilution for all), and glyceraldehyde 3-phosphate dehydrogenase (GAPDH; 1:25,000dilution) overnight at 4°C and then incubated with the horseradish peroxidase–conjugated secondary antibody (Jackson Immuno Research Laboratories, West Grove, PA, USA) for 1 hour. Next, the proteins were visualized using enhanced chemiluminescence (ECL; Millipore). The Western blotting images were acquired using the UVP BioSpectrum 500 imaging system and examined using Vision Works LS Analysis software (Upland, CA, USA). The expression level of GAPDH was used as a loading control.

### Immunocytochemistry

After 24 hours, the ARPE-19 cells were seeded into 8-well cell chamber slides and transfected with miRNA (mimic/inhibitor). Next, they were incubated for up to 24 hours in the presence or absence of TGF-β2. After the cells were washed three times with PBS, they were fixed with 4% paraformaldehyde and then treated with 0.1% Triton X-100 for 10 min on ice. They were then incubated with 5% bovine serum albumin in PBS for 1 hour at room temperature. Anti-ZO-1 (1:200dilution; Zymed Laboratories) and antivimentin antibodies (1:200 dilution; Santa Cruz Biotechnology) were used as the primary antibodies. The secondary antibodies comprised DyLight 488 antirabbit immunoglobulin G (IgG) and DyLight 594 antimouse IgG antibodies (1:200 dilution; Bethyl Laboratories, Montgomery, TX, USA), respectively. The nuclei were counterstained with 4’,6’-diamidino-2-phenylindole (Sigma-Aldrich). The preparation was fixed with 70% glycerol and examined using a fluorescence microscope (CKX41, Olympus Corporation, Tokyo, Japan).

### Scratch wound healing assay

A modified in vitro scratch test is used to assess cell migration in vitro. In brief, ARPE-19 cells at 95% confluence were serum starved for 24 hours and treated with 10 μg of mitomycin-C for 2 hours. A scratch wound was then inflicted on the monolayer using a sterile 200-μL pipette tip. Next, the medium was removed and replaced with fresh serum-free medium containing the test substance or control. An Olympus IX70 microscope equipped with a digital camera was used to capture photographs in the selected area at 24 and 48 hat 4× magnification. The width of the scratch was measured by calculating the distance between its two edges using Image J software.

### Cell migration assay

A modified Boden’s chamber assay was also used to evaluate cell migration. In brief, ARPE-19 cells were seeded in the upper chamber of a 24-well plate that was coated with fibronectin and had pores8μmin diameter at a density of 5×10^4^ cells/well (Corning Incorporated, Corning, NY, USA). The lower chamber was seeded with 0.1% FBS–DMEM–F12 containing 10 ng/mL TGF-β2. After 5 hours of incubation, the cells that migrated to the lower side (insert) were washed with PBS, fixed with cold methanol (4°C) for 10 min, and then counterstained with hematoxylin (ThermoScientific) for 20 min. The number of migrated cells was counted using phase-contrast microscopy. Four randomly selected fields were counted for each insert.

### Collagen gel contraction assay

Collagen gel contraction was assessed with some modifications. In brief, rat tail type I collagen (Sigma-Aldrich) was dissolved in 0.1% acetic acid in sterile distilled water and stored overnight at 4°C. The 24-well plate was coated with 2% FBS overnight to block nonspecific binding. The ARPE-19 cells (1.0×106 cells/mL) were resuspended in serum-free DMEM-F12. The cell suspension was mixed with 5.0 mL of 3 mg/mL type I collagen, 3.0 mL of concentrated serum-free DMEM-F12 containing glutamine and antibiotics, and 391 μL of 1 mM NaOH. Next, 350 μL of the mixture was added to each FBS-coated well and was allowed to solidify through incubation in 5% CO_2_ at 37°C for 1 hour. After 1.5 hours, the collagen gels were removed from the bottom of the wells using a microspatula, and 1 mL of 10% FBS–DMEM–F12 was added on top of each gel. After 24 hours, the medium was removed, and the gels were washed with serum-free DMEM–F12 and cultured in serum-free DMEM–F12 containing 10ng/mL TGF-β2 at 37°C for another 3 days. The cell media were changed every other day. Collagen gels without RPE cells were used as the baseline data for contraction. On the third day, a LAS-3000 charge-coupled device camera (Fujifilm, Dusseldorf, Germany) was used to observe, record, and measure the surface area of each matrix. The percentage of gel contraction was calculated as follows: [(gel size at day 1 − gel size at day 3)/gel size at day 1] ×100. All experiments were conducted at least three times.

### Luciferase reporter assay

The putative binding sites of miR-1285 in the 3′-UTR of Smad4 or complementary DNA fragment and the mutated 3’-UTR of Smad4 were amplified and subcloned into pGL3 Luciferase Reporter Vectors (Promega, Madison, WI, USA). The cells were seeded into 6-well plates at a density of 1 × 10^5^ cells/well and then co-transfected with the vectors (containing the original or mutated 3′-UTRof Smad4) and miR-1285. After 24 hours of incubation, the cells were harvested and prepared using reporter lysis buffer. Next, the luciferase activity in each well was measured using the dual-luciferase reporter assay system (Promega Corp, Madison, WI, USA).

### Ethics statement

Five New Zealand albino rabbits weighting 1.5 kg each were used for this study. The animal study was approved by and carried out in accordance with and the Institutional Animal Care and Use Committee (accredited by the Association for Assessment and Accreditation of Laboratory Animal Care International), National Defense Medical Center, Taipei, Taiwan (No: IACUC-20-169). The rabbits received humane care as outlined in the Guide for the Care and Use of Laboratory Animals [23]. They were kept under standard condition (20±11°C, 12 hours light/12 hours dark cycles) with the floor area of 1.5 ft^2^ and height of 16 in. Adequate supplies of food and fresh water devices were provided in sufficient numbers to allow ready access for rabbits. Daily observation and activity check of the rabbits was performed during the experiment. All animal experiments were performed in compliance with the Association for Research in Vision and Ophthalmology Statement for the Use of Animals in Ophthalmic and Vision Research.

### In vivo inhibition of PVR progression by miR-1285 in a rabbit model of PVR

Following pupillary dilation with instillation of eye drops (two drops each) containing 5% phenylephrine and 1% tropicamide, the rabbits were anesthetized with 50 mg/kg Zoletil and 10 mg/kg xylazine. A 31-guage needle was passed through the sclera 2.5 mm behind the scleral limbus, and 0.1 mL of 10 ng/mL TGF-β2 and 0.1 mL of balanced salt solution (BSS) containing approximately 1 × 10^6^ MiR-1285 cells or ARPE-19 cells transfected with miR-NC was injected directly over the optic disc of the right eye. A total of four doses were administered to each eye on days 1, 8, 15, and 22.

After the injection, the rabbits were examined by indirect ophthalmoscopy twice a week for 4 weeks (days 1–29). PVR progression was determined using the Fastenberg classification. The staging criteria are listed in S1 Table. The euthanasia of rabbits was then performed and conducted in a CO2 chamber after the experiment. After CO2 exposure, the rabbits were placed in room air for 20 min to allow for possible recovery [24].

### Histochemical staining

All rabbit eyes were refixed in neutral-buffered formalin, paraffin embedded, and sectioned (5-μm thickness). The serial sections closest to the defect center were harvested, left to float in a water bath at 40°C, placed on organosilane-coated (silanized) microscope slides, and baked overnight at 37°C. The sections were deparaffinized in xylene and rehydrated in serial ethanol rinses for hematoxylin and eosin staining.

### Statistical analysis

Data are presented as means ± standard errors of the mean. All experiments were performed in triplicate and repeated at least three times. The data were analyzed using GraphPad Prism for Windows version 5.01 (GraphPad Software, La Jolla, CA, USA). One-way analysis of variance was performed to determine statistically significant differences, followed by Tukey’s post hoc test. A p value of <0.05 was considered statistically significant.

## Results

### Expression of miR-1285 was suppressed by TGF-β

**Fig 1:**
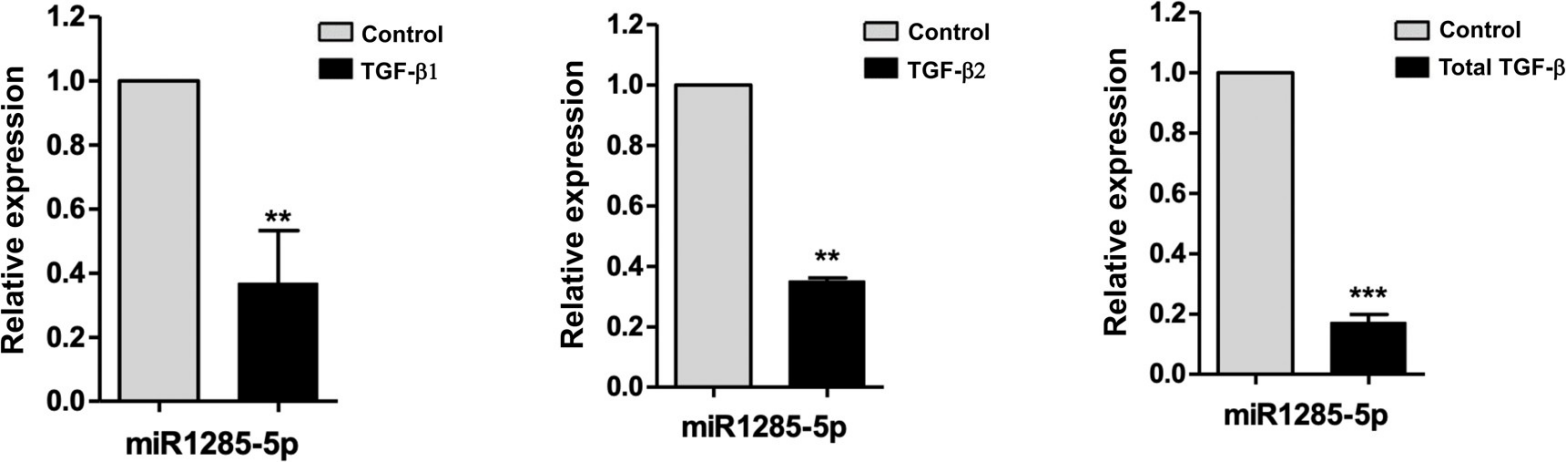
Expression of microRNA (miR)-1285 was suppressed by TGF-β. (A) (B) (C) Expression of miR-1285 in ARPE-19 cells incubated in the presence or absence of transforming growth factor (TGF)-β1, TGF-β2, and total TGF-β (10 ng/mL) for 48 h. The expression levels of miR-1285 were normalized to those of the control, U6 small nuclear RNA. Data are presented as the means ± standard errors of the mean of three replicates. **p< 0.01; ***p<0.001.

TGF-β, multifunctional cytokine, acts as a potent inducer of the EMT in RPE cells. Studies have demonstrated this induction is implicated in the pathogenesis of PVR [25–28]. Choudhury et al. indicated that miR-1285 plays a key role in the functions of RPE cells [28]. Therefore, we first evaluated the association between TGF-β stimulation and miR-1285 expression in ARPE-19 cells, which showed that miR-1285 expression was significantly reduced after treatment with TGF-β1, TGF-β2, and TGF-β ligands (Fig 1). Concentrations of TGF-β2 were higher than those of the other two isoforms of TGF-β, and TGF-β2 concentration and contractility correlate strongly [29, 30]. Therefore, TGF-β2 (10 ng/mL) was used in the subsequent experiments.

### RPE transdifferentiation was inhibited by miR-1285 delivery

**Fig 2:**
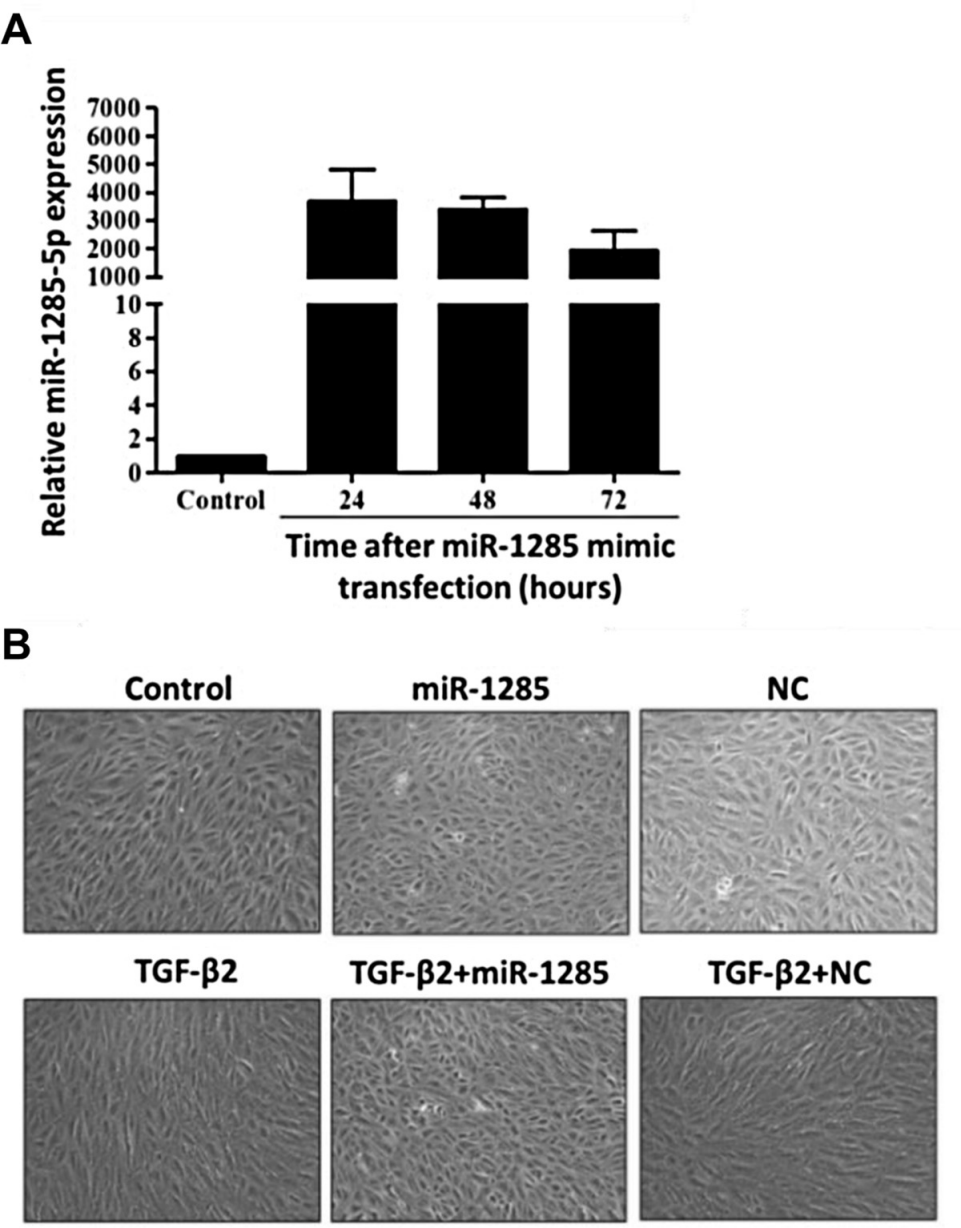
Transdifferentiation in retinal pigment epithelial cells was inhibited by microRNA (miR)-1285 delivery. (A) The expression levels of miR-1285 in ARPE-19 cells transfected with negative control or a miR-1285 mimic, as determined through a real-time quantitative polymerase chain reaction. (B) Morphologic alterations in ARPE-19 cells transfected with negative control or miR-1285 and incubated in the presence or absence of TGF-β2 (10 ng/mL). The cells were examined by phase-contrast microscopy.

The ARPE-19 cells were transfected with miR-1285 to determine transfection efficiency. RT-qPCR revealed that the level of miR-1285 in the cells was 4000 times higher than that in cells transfected with NC at the 24-hour time point. The expression levels decreased gradually over time but remained 2000 times higher than that of cells transfected with NC even at the 72-hour time point (Fig 2A). Furthermore, TGF-β2 induced the fibroblastic transition of the ARPE-19 cells; however, as shown in Fig 2B, miR-1285 inhibited the progression of these alterations (Fig 2B). Cells incubated in the absence of TGF-β2 and transfected with miR-1285 displayed an epithelial morphology.

### Biomarker expression of the EMT in TGF-β2-treated ARPE-19 cells through miR-1285 delivery

**Fig 3:**
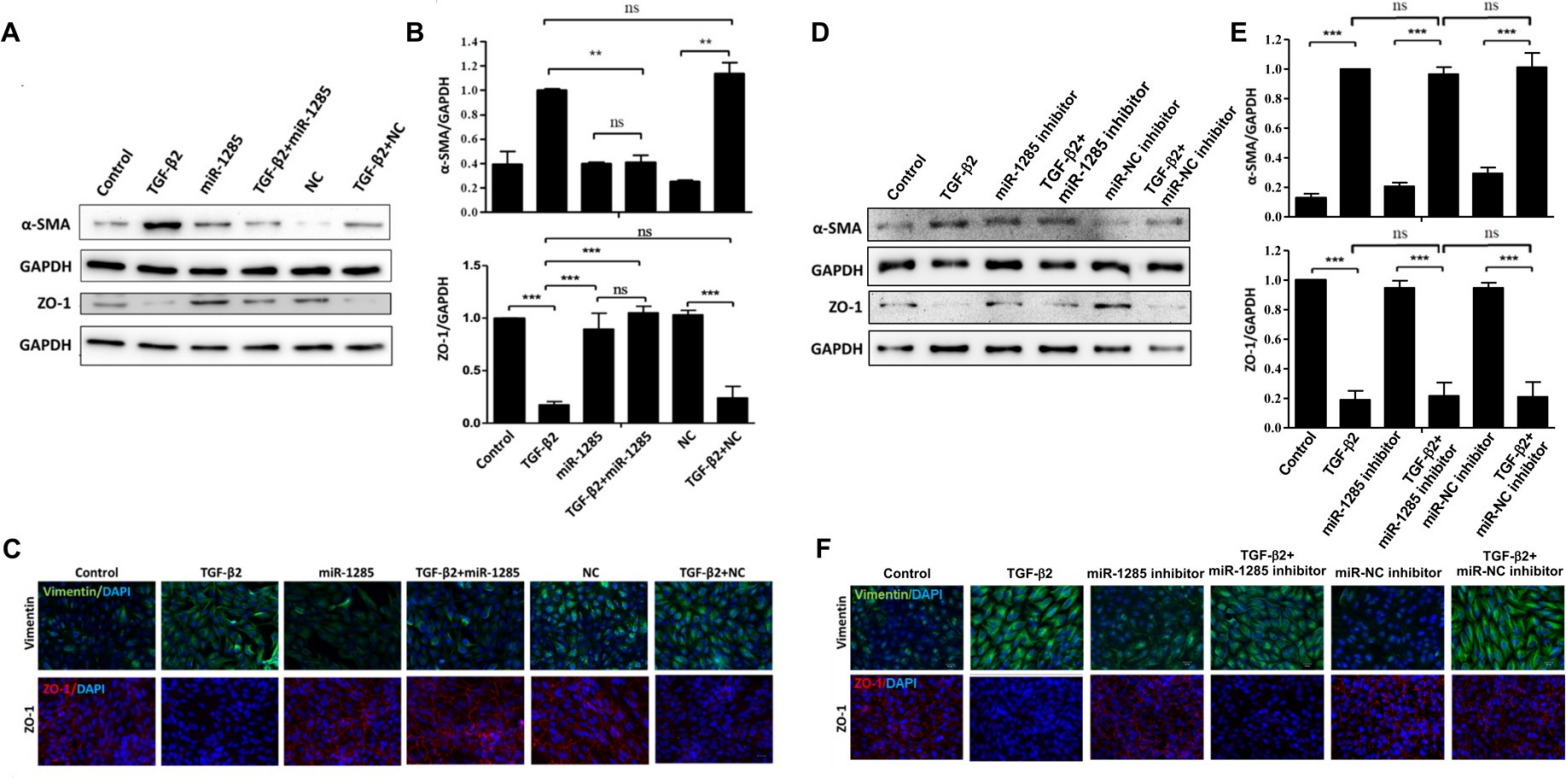
Biomarker expression of the epithelial–mesenchymal transition (EMT)in ARPE-19 cells treated with TGF-β2 through microRNA (miR)-1285 delivery. (A) Immunoblot analyses of the effects of miR-1285 overexpression on the expression of the fibroblastic and epithelial factors, α-smooth muscle act in and zonula occludens-1 (ZO-1), during TGF-β2-induced EMT in the ARPE-19 cells. (B) Quantification of the immunoblot data shown in (A). Three independent experiments were performed. Data are presented as the means± standard errors of the mean. **p<0.01; ***p<0.001; ns: not significant according to an independent one-way analysis of variance. (C) Representative images of immunostained fibroblastic (vimentin) and ZO-1 in ARPE-19 cells transfected with negative control or an miR-1285 mimic and treated with TGF-β2 for 48h. Nuclei were stained with 4′,6-diamidino-2-phenylindole. (D) Immunoblot analyses of the effects of miR-1285 inhibitor on the expression of fibroblastic and epithelial factors, α-smooth muscle actin and zonula occludens-1 (ZO-1), during TGF-β2-induced EMT in ARPE-19 cells. (E) Quantification of the immunoblot data shown in (D). Three independent experiments were performed. Data are presented as the means ± standard errors of the mean. **p<0.01; ***p<0.001; ns: not significant according to an independent one-way analysis of variance. (F) Representative images of immunostained fibroblastic (vimentin) and ZO-1 in ARPE-19 cells transfected with negative control or an miR-1285 inhibitor and treated with TGF-β2 for 48 h. Nuclei were stained with 4′,6-diamidino-2-phenylindole.

To evaluate the effects of transfection of ARPE-19 cells with an miR-1285 mimic on RPE cells in which TGF-β2 induced the EMT, we used Western blotting to examine the protein expression of ZO-1 and α -SMA (epithelial and mesenchymal markers, respectively). As shown in Fig 3A and 3B, quantitative immunoblot analyses revealed that, compared with the cells transfected with the NC, the TGF-β2-treated ARPE-19 cells had significantly higher levels of α-SMA and significantly lower levels of ZO-1, representing epithelial proteins. Furthermore, the cells transfected with miR-1285 after TGF-β2 treatment exhibited reduced α-SMA expression and increased ZO-1 expression; these results were contrary to those observed in the cells transfected with the NC. Immunocytochemistry analysis—specifically immunofluorescence—was used to examine the effects of transfection of ARPE-19 cells with NC or the miR-1285 mimic on the expression of ZO-1 and vimentin (another mesenchymal marker), in the ARPE cells treated with TGF-β2. Consistent with the Western blotting results, ZO-1 and vimentin expression was lower and higher in those cells, respectively, than in the cells transfected with NC. In contrast, in the TGF-β2-treated ARPE-19 cells, the cells transfected with miR-1285 mimic downregulated and upregulated vimentin and ZO-1, respectively (Fig 3C). However, the expression of these EMT-related proteins was not affected in cells treated with miR-1285 inhibitor (Fig 3D–3F). Moreover, miR-1285-5p expression was lower than the expression of other well-known microRNAs (miR-125, miR-222, miR-100, and miR-185) (S1 Fig). This indicated that miR-1285 inhibitor did not affect the expression of EMT-related proteins due to low expression of endogenous miR-1285 in APRE-19 cells. Taken together, these results demonstrate that miR-1285 transfection suppressed TGF-β2-induced EMT in the ARPE-19 cells.

### Migration of TGF-B2-treated ARPE19 is passivated by miR1285 delivery

**Fig 4:**
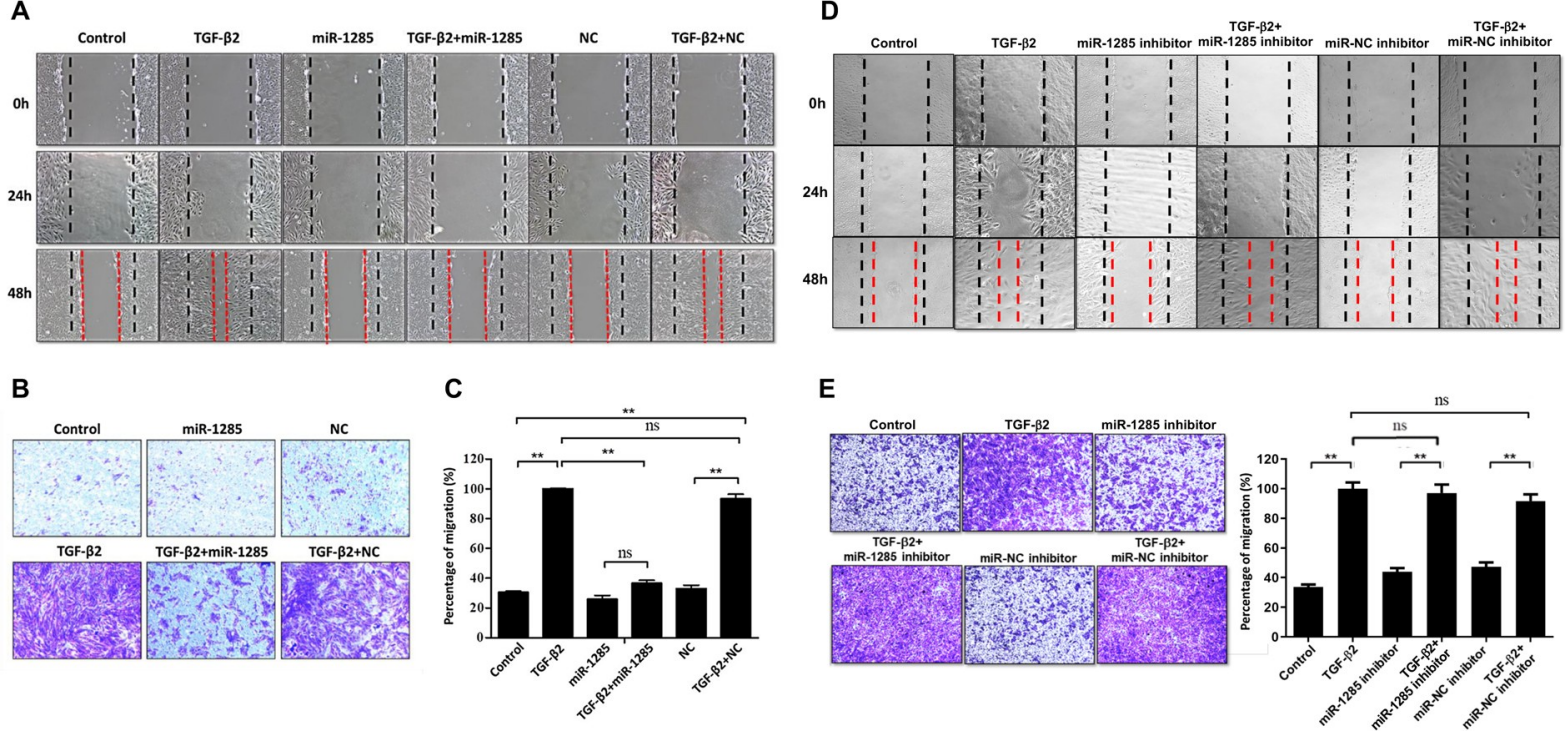
Migration of the ARPE-19 cells treated with TGF-β2 was passivated by microRNA (miR)-1285 delivery. (A) Confluent cells were transfected with negative control or a miR-1285 mimic. A scratch was inflicted to the cell surfaces, and then the cells were treated with or without TGF-β2. Light microscopy images were taken at 0, 24, and 48h after scratch infliction. The control panel shows the untreated cells. The dashed lines delineate the central observation area and were added by a masked observer to help clarify the extent of migration. Magnification: 4×. The data are representative of at least three independent experiments. (B) After the ARPE-19 cells were transfected with the miR-1285 mimic for 24h, they were allowed to migrate through the filter of Transwell chambers for 5 h with or without TGF-β2 (10 ng/mL) and stained with crystal violet. (C) The number of cells that migrated was quantified by counting the number of cells appearing in the lower chamber in four independent vision fields with a 20× microscope objective. Results are presented as the means ± standard errors of the mean of three independent experiments. (D) Confluent cells were treated with negative control or an miR-1285 inhibitor. A scratch was inflicted to the cell surfaces, and then the cells were treated with or without TGF-β2. Light microscopy images were taken at 0, 24, and 48h after scratch infliction. The control panel shows the untreated cells. The dashed lines delineate the central observation area and were added by a masked observer to help clarify the extent of migration. Magnification: 4×. The data are representative of at least three independent experiments. (E) After the ARPE-19 cells were treated with the miR-1285 inhibitor for 24h, they were allowed to migrate through the filter of Transwell chambers for 5 h with or without TGF-β2 (10 ng/mL) and stained with crystal violet. The number of cells that migrated was quantified by counting the number of cells appearing in the lower chamber in four independent vision fields with a 20× microscope objective. Results are presented as the means ± standard errors of the mean of three independent experiments. **p<0.01; ns: not significant.

TGF-β2-induced EMT in RPE cells is an initiating event in many fibrotic processes, including collagen contraction, cell migration and proliferation, which occur during PVR pathogenesis. Therefore, we studied the effects of miR-1285 transfection on cell migration in TGF-β2-treated ARPE-19 cells using a scratch wound healing assay. Cells were serum starved for 24 hours and pretreated with 10 μg of mitomycin-C for 2 hours to suppress cell proliferation. A scratch was inflicted on the cell surfaces, and the cells were observed at 0, 24, and 48 hours with or without TGF-β2. Treatment with TGF-β2 alone enhanced wound closure in a time-dependent manner. At 48 hours, miR-1285 transfection had completely suppressed TGF-β2-induced wound closure (Fig 4A). In sum, miR-1285 transfection inhibited wound closure in TGF-β2-treated ARPE-19 cells. Because cell proliferation and migration are essential to wound closure, we used mitomycin-C to suppress cell proliferation. Therefore, the results suggest that miR-1285 transfection inhibited TGF-β2-induced wound closure by suppressing cell migration (Fig 4A).

To reiterate, miR-1285 transfection attenuated the positive effects of cell migration on wound closure in the TGF-β2-treated ARPE-19 cells. Next, we used a modified Boyden chamber method to quantify cell migration in the ARPE-19 cells. As shown in Fig 4B and 4C, compared with that in the NC cells, cell migration was significantly promoted and suppressed in the ARPE-19 cells, respectively, under TGF-β2 stimulation and miR-1285 transfection (*p*<0.01). No significant differences were noted between the vehicle control and NC groups. In addition, cell migration was not affected in cells treated with miR-1285 inhibitor (Fig 4D and 4E). In short, the results indicate that miR-1285 transfection inhibited TGF-β2-induced cell migration in the ARPE-19 cells.

### Effects of miR-1285 on the EMT are exerted through Smad4

**Fig 5:**
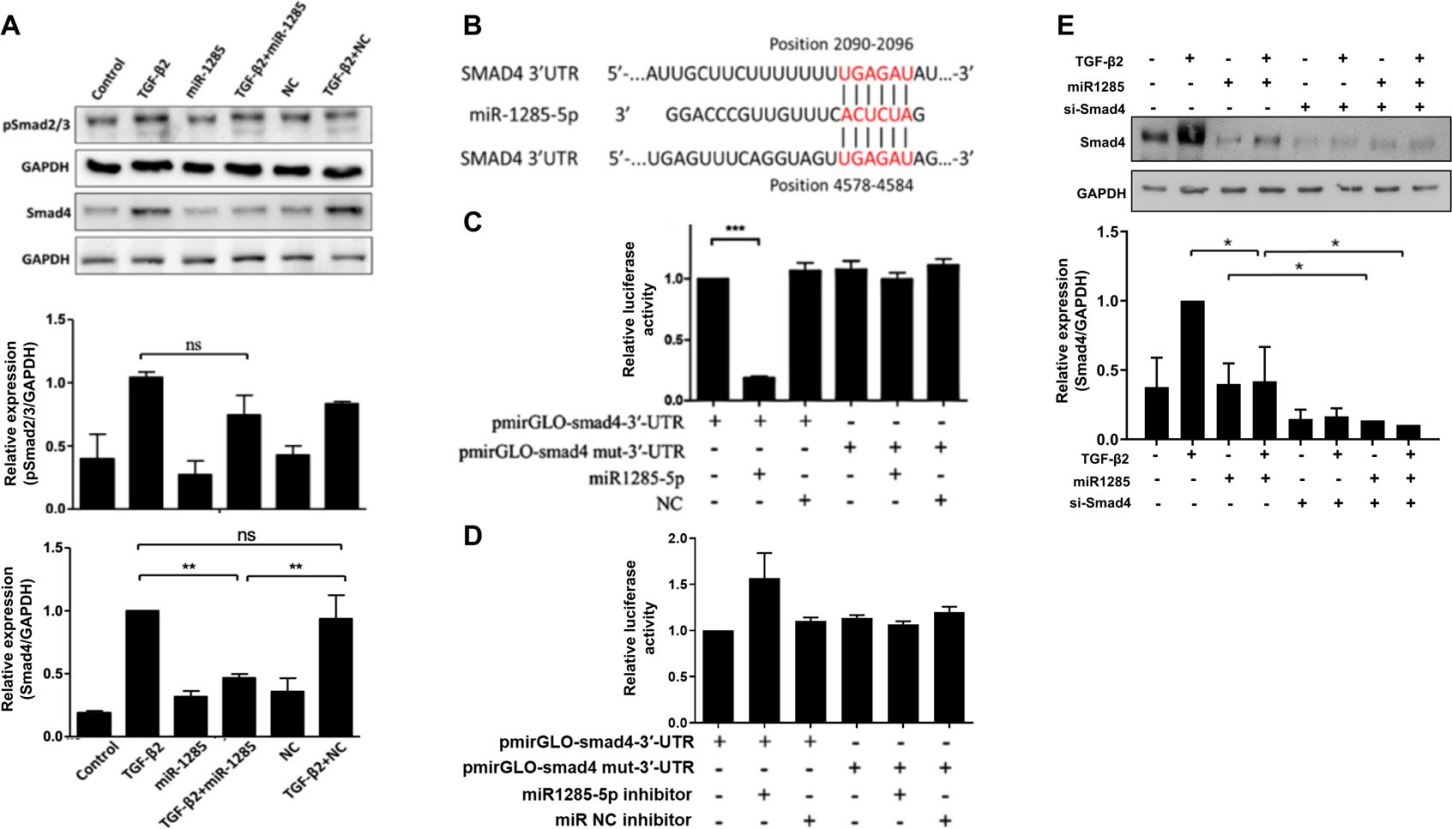
Micro RNA (miR)-1285 exerted effects on the EMT through Smad4. (A) Representative images and quantification of immunostained phosphorylated Smad2/3 and total Smad4 in the TGF-β2-treated ARPE-19 cells transfected with negative control (NC) or miR-1285. Data are presented as the means ± standard errors of the mean of three replicates. Independent one-way analysis was performed, followed by Tukey’s post hoc test. (B) Putative miR-1285 seed matches within the 3′ untranslated region (3′-UTR) of Smad4. The web server TargetScan predicted two perfect seed matches that were conserved across various species. (C) Relative luciferase activity of a pGL3 luciferase reporter vector fused to the native or mutated 3′-UTRof Smad4 in ARPE-19 cells cotransfected with NC or the miR-1285 mimic. (D) Relative luciferase activity of a pGL3 luciferase reporter vector fused to the native or mutated 3′-UTRof Smad4 in ARPE-19 cells co-treated with NC or the miR-1285 inhibitor. (E) Representative images and quantification of immunostained Smad4 in the TGF-β2-treated ARPE-19 cells transfected with negative control (NC) or miR-1285 inhibitor. Data are presented as the means ± standard errors of the mean of three replicates. Independent one-way analysis was performed, followed by Tukey’s post hoc test. *p< 0.05; ***p< 0.001; ns, not significant.

The major signaling pathway in the TGF-β family involves the phosphorylation of Smad proteins by serine/threonine kinase receptors. A study indicated that TGF-β/Smad signaling is required for EMT of RPE cells. Moreover, miR-1285 was shown to affect the proliferation and metastasis of non-small-cell lung carcinoma cells by downregulating Smad4, a central mediator of TGF-β intracellular signaling [27]. Taking this evidence into account, we suppressed the Smad pathway to determine whether miR-1285 transfection affected the EMT in the ARPE-19 cells. Western blotting analysis was used to assess the effects of miR-1285 transfection on the phosphorylation of Smad 2/3 and Smad4 in the TGF-β2-treated ARPE-19 cells. As shown in Fig 5A, in the presence of 10 ng/mL TGF-β2, cells transfected with a miR-1285 mimic had lower levels of Smad4 than those transfected with NC. No effects were observed on Smad2/3phosphorylation. These results were confirmed through quantitative analyses of immunoblotting assays, which indicated that miR-1285 significantly affected Smad4 expression.

To identify putative regulatory targets of miR-1285, we performed in silico analysis using the web server Target Scan. A search of the 3′-UTRof the Smad4 gene revealed two evolutionary highly conserved target sequences of miR-1285 (Fig 5B). Next, a luciferase reporter assay was performed to determine whether the 3′-UTRof was an actual target of miR-1285 in ARPE-19 cells. In cells co-transfected with the reporter plasmid, miR-1285 overexpression reduced luciferase activity by approximately 85%. However, the luciferase activity of a pGL3luciferase reporter vector fused to the native or mutated 3′-UTRof Smad4 in ARPE-19 cells co-transfected with the NC or the miR-1285 mimic remained the same (Fig 5C). In addition, the luciferase activity was not induced in cells treated with miR-1285 inhibitor (Fig 5D). SMAD4 siRNA significantly increased effects of miR-1285 overexpression on repression of TGF-enhanced SMAD4 expression (Fig 5E). Taken together, these results indicate that miR-1285 transfection inhibited TGF-β2-induced EMT and PVR development in RPE cells, at least in part, through suppression of the Smad4 signaling pathway.

### PVR is inhibited by miR-1285 overexpression in vitro

**Fig 6:**
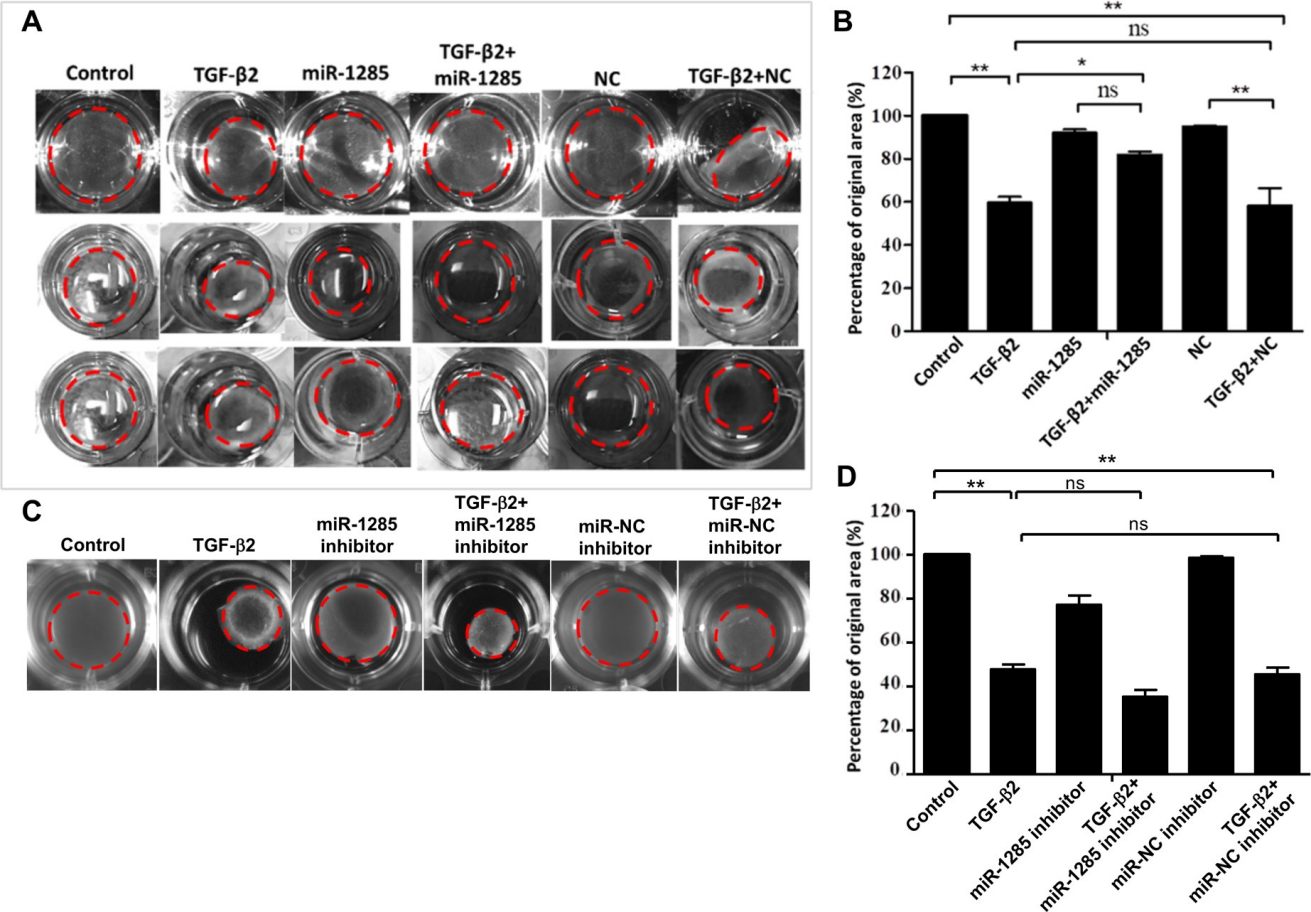
PVR was inhibited by microRNA (miR)-1285 overexpression in vitro. (A) ARPE-19 cells were pretransfected with negative control or the miR-1285 mimic for 24h and then treated with 10 ng/mL TGF-β2 in the presence or absence of the miR-1285 mimic for 3 days. (B) Contraction or expansion was determined as a percentage of the original area Results are presented as the means ± standard errors of the mean of three independent experiments. (C) ARPE-19 cells were pretreated with negative control or the miR-1285 inhibitor for 24h and then treated with 10 ng/mL TGF-β2 in the presence or absence of the miR-1285 inhibitor for 3 days. (D) Contraction or expansion was determined as a percentage of the original area Results are presented as the means ± standard errors of the mean of three independent experiments. *p<0.05; **p<0.01; ns: not significant.

Given study reports showing that TGF-β2-induced collagen gel contraction plays a vital role in the pathology of PVR, we posited that any inhibition of TGF-β2 would strongly suppress collagen gel contraction in PVR. Therefore, we tested the effects of miR-1285 transfection on collagen gel contraction in TGF-β2-treated ARPE-19 cells using a collagen matrix contraction assay. Specifically, freshly polymerized collagen matrices containing ARPE-19 cells were assessed for collagen gel extraction. In the presence of TGF-β2, the matrices shrunk to approximately 60% of the area of the untreated gel; however, this shrinkage was attenuated by the overexpression of miR-1285 (Fig 6A). This result (with contraction or expansion defined as a percentage of the original area) was statistically significant across all three independent experiments conducted (Fig 6B). However, the development of PVR was not affected in cells treated with miR-1285 inhibitor (Fig 6C and 6D). These results suggest that miR-1285 transfection attenuated the effect of collagen gel contraction in the TGF-β2-treated ARPE-19 cells.

### MiR-1285 inhibits PVR development in vivo

**Fig 7:**
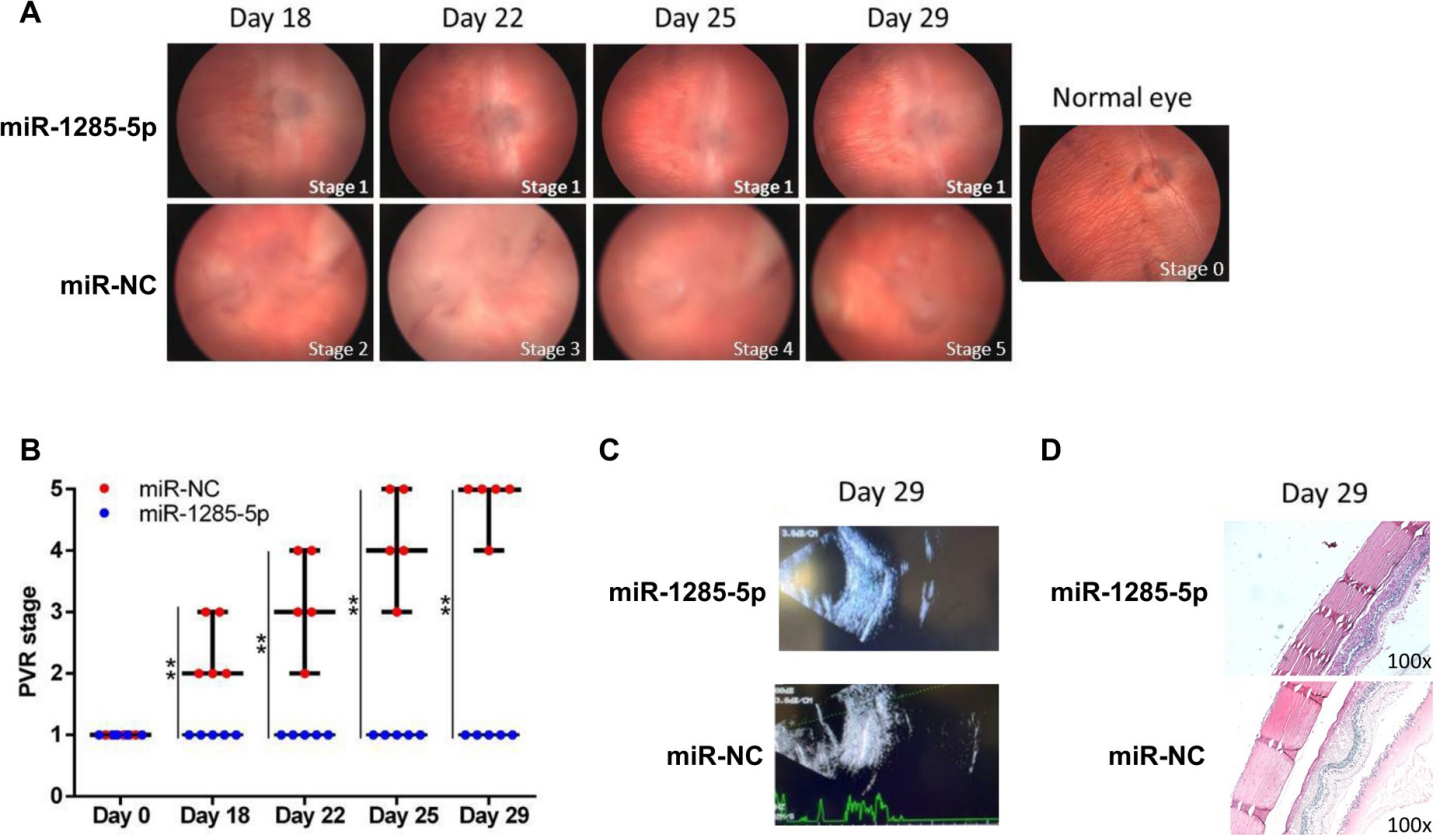
MicroRNA (miR)-1285 inhibited the development of proliferative vitreoretinopathy (PVR) in vivo. (A) 0.1 mL of 10 ng/mL TGF-β2 and 0.1 mL of balanced salt solution containing approximately 1 × 10^6^miR-1285 and ARPE-19 cells transfected with miR-negative control (NC) were injected into the right eyes. A total of four doses was administered to each eye on days 1, 8, 15, and 22. PVR progression was determined according to the Fastenberg classification (stages 1–5) at day 29 postinjection. (B) Results from the independent samples *t* test of differences in PVR stage between the control group and the miR-1285-treated group. (C)Ultrasonic examination of the control eye and the miR-1285-treated eye at day 29 post injection. (D)Hematoxylin and eosin staining revealed the presence of dense fibrovascular membrane tissue and considerably higher infiltration of inflammatory cells on the retinal surface of eyes treated with miR-NC compared with that of eyes treated with miR-1285.

Examination of the in vivo effects of miR-1285 on PVR development revealed that 0.1 mL of 10 ng/mL TGF-β2 and 0.1 mL of BSS containing approximately 1 × 10^6^ ARPE-19 cells transfected with miR-1285 and miR-NC were injected into rabbit right eyes. A total of four doses were administered to each eye on days 1, 8, 15, and 22. As shown in Fig 7A, in the control group, severe retinal detachment (stages 4 and 5 in the Fastenberg classification) was observed in all rabbits 29 days after injection. In contrast, PVR progression was attenuated in all rabbits (stage 1 in the Fastenberg classification). S2 Table summarizes the disease status of the rabbits (staged using the Fastenberg classification). As shown in Fig 7B, miR-1285 significantly inhibited PVR development. Ultrasound reports revealed severe retinal detachment in the eyes of the control rabbits. The rabbits treated withmiR-1285-5p did not develop this condition (Fig 7C).

Hematoxylin and eosin staining indicated the presence of dense fibrovascular membrane tissue and considerably higher infiltration of inflammatory cells on the retinal surface of eyes treated with miR-NC compared with that of eyes treated with miR-1285 (Fig 7D). Comparison with the eyes injected with ARPE-19 cells transfected with miR-NC showed that PVR progression was significantly inhibited in the eyes injected with ARPE-19 cells transfected with miR-1285. This indicates that miR-1285 has the potential to prevent and treat PVR.

## Discussion

**Fig 8:**
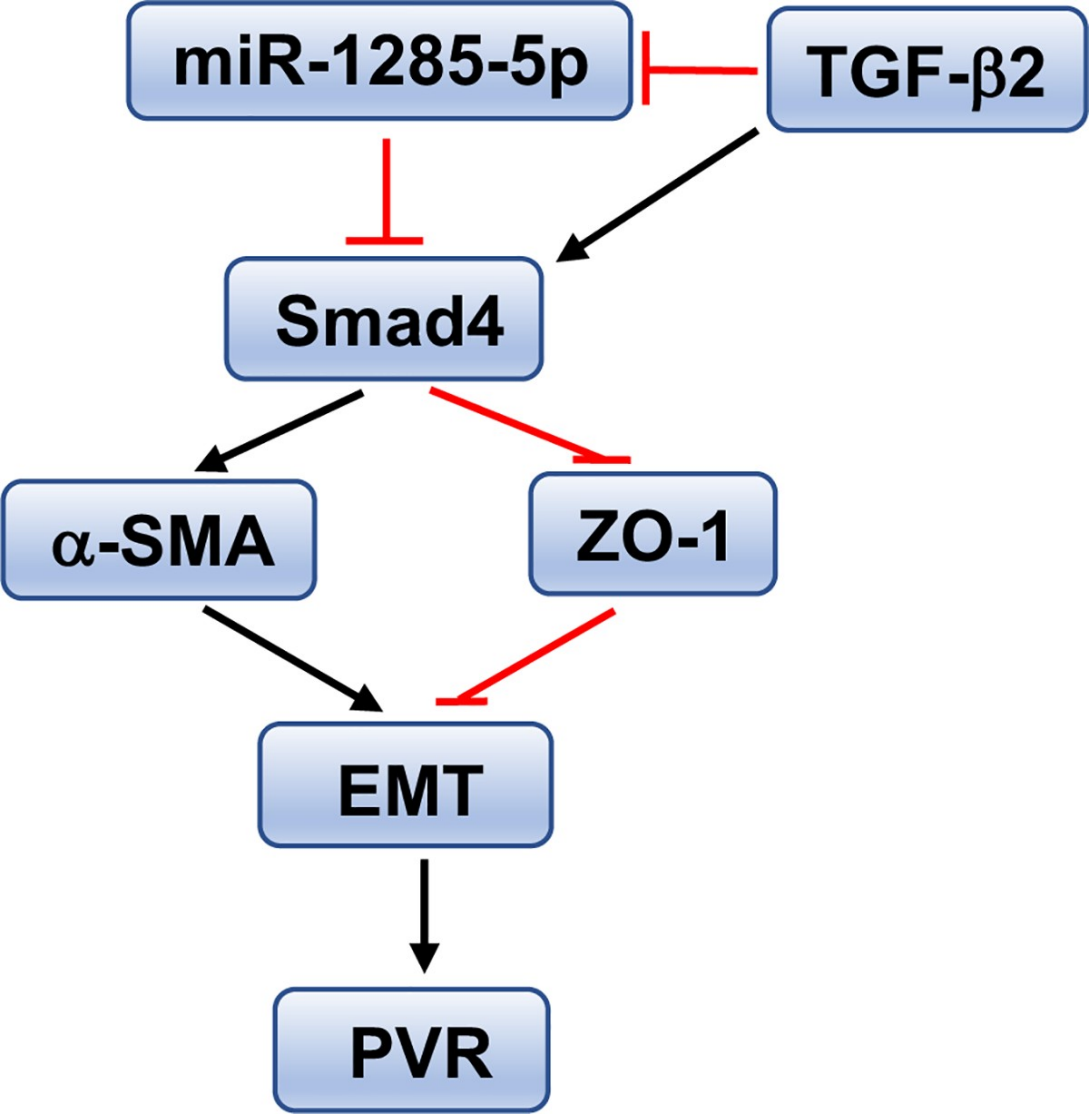
A proposed model of the mechanistic interaction of Smad4 and miR-1285 involved in TGF-β-induced EMT and development of PVR.

The present study has demonstrated that overexpression of miR-1285 attenuates TGF-β2-induced EMT in a rabbit model of PVR, and the effect of miR-1285 in PVR is dependent on Smad4. PVR is formed when the vitreous is in contact with the retinal pigment epithelium and tightly connected to RPE cells by contacting the monolayer of pigmented cells through extension [31]. Generally, RPE cells remain quiescent, maintaining their characteristic morphology and function. The external BRB prevents the fluid in the choroidal blood vessels from entering the retina. In the case of retinal rupture, BRB ruptures, and RPE cells are exposed to and activated by various growth factors and cytokines in the vitreous. This results in the cells undergoing EMT, which is a process of morphological and phenotypic trans-differentiation, and is the main pathological process involved in PVR [32, 33]. During EMT, the morphology and phenotype of epithelial cells transform, becoming similar to those of mesenchymal cells [34]. The morphological feature of this trans-differentiation is to reduce cell adhesion and increase cell migration and invasion. Phenotypic changes include decreased expression of epithelial markers (such as ZO-1) and increased expression of mesenchymal markers (such as α-SMA and vimentin) [26]. The essential polypeptide ZO-1 is found in the TJ complex, and the structure of TJ is essential for maintaining the normal structure or function of epithelial cells [33, 35]. Vimentin and α-SMA are both intracellular cytoskeletal proteins, with α-SMA participating in cell movement and contraction, and vimentin playing a key role in stabilizing cell structure during cell migration [26]. Recent studies have revealed that miRs can regulate TGF-β signaling during EMT [36, 37]. As shown in Fig 8, the present study demonstrated that miR-1285 expression was downregulated in ARPE-19 cells treated with TGF-β1 and TGF-β2. Moreover, miR-1285 overexpression led to the upregulation of ZO-1 and the downregulation of α-SMA actin and vimentin in ARPE-19 cells treated with TGF-β2. These results indicate that miR-1285 suppresses phenotypic changes in cells by mediating intracellular cytoskeletal proteins and epithelial protein markers, and that it can further prevent EMT remodeling.

A recent study indicated that RPE cells converted to myofibroblast phenotypes acquire migratory properties and participate in the formation of the epiretinal membrane following the clinical evolution of PVR [32]. Therefore, inhibiting the migration of RPE cells as functional myofibroblasts to the retina is a potential therapeutic target for PVR [38–40]. It is well known that EMT is regulated by numerous factors, including inflammatory cytokines and growth factors. In our previous study on age-related macular degeneration, a miRNA array revealed that miR-1285 was downregulated in ARPE-19 cells treated with the inflammatory cytokine TNF-α; TNF-α is one of the most prominent inflammatory cytokines, and its mRNA and proteins are widely expressed in PVR membranes. Takahashi et al. state that TNF-α is mainly derived from activated macrophages, and RPE and glial cells in PVR membranes also release it [41]. In vitro and in vivo models both provided evidence that the proinflammatory cytokine TNF-α plays a key role in the induction of fibrosis associated with the mesenchymal change of RPE cells. These authors also showed that there is interplay between TNF-α and TGF-β2 signaling, which induces the EMT in ARPE-19 cells [41]. Other studies also have reported that ARPE-19 cells undergo EMT through activation of the mitogen-activated protein kinase and Smad pathways arising from the stimulation of both TNF-α and TGF-β2, and then they migrate and form aggregates [41, 42]. Yoshimatsu et al. indicated that TGF-β was more highly expressed in human endothelial cells treated with a combination of TGF-β and TNF-α than in cells cultured with either of these cytokines, suggesting cooperation between TGF-β and TNF-α in augmenting TGF-β signals [43]. In the present study, miR-1285 attenuated the acquisition of migratory properties in ARPE-19 cells treated with TGF-β2. In addition, significant effects were noted on the inhibition of wound closure and cell migration.

The critical point in the pathogenesis of PVR is the contraction of cell membranes, which promotes retinal detachment and, in turn, leads to vision loss [44]. Collagen gel contraction is the classic model for studying wound healing [45], a process in which the main cell types are fibroblasts and transdifferentiated RPE cells [46–48]. In the present study, possibly because of reduced α-SMA and vimentin expression and increased ZO-1 expression, miR-1285 reduced collagen gel contraction in ARPE-19 cells treated with TGF-β2. In short, miR-1285 may reduce the contraction of fibrotic cellular membranes in the pathogenesis of PVR.

As small non-coding RNAs, miRs bind to complementary sequences on target messenger RNAs, which silences them. These miRs are associated with the occurrence, development and metastasis of various cancers [10]. Based ib the background and type of tumor, miR-1285 can serve as either a tumor suppressor or cancer promoter [49–51]. These seemingly contradictory effects are shown to be the result of differential gene expression patterns and, for specific miRs, are part of the tumor-specific interactome. Few studies have shown that miRs act on the retina or RPE cells [12–15]. The role of miR in the progression of PVR, especially in the EMT of RPE cells, which is the main mechanism of RVR cell development, has not been studied extensively. Identifying the role of miRs in EMT of RPE cells in vitro and PVR in vivo may help to gain a deeper understanding of gene expression associated with EMT. Identifying a set of miRs and their target genes for the same disease may hold promise for exploring the possibility of miR-based treatments.

TGF-β is overexpressed in the vitreous of patients with proliferative diabetic retinopathy and PVR. It is also expressed in the proliferative membranes of individuals with these diseases [52]. In the present study, we found that after treatment with TGF-β1, TGF-β2 and TGF-β ligand, the expression of miR-1285 in ARPE-19 cells was significantly reduced. The main TGF-β isoform TGF-β2 in the eye is detected primarily in the posterior segment of the eye and aqueous humor [53]. Notably, abnormally high expression of TGF-β2 in the ERMs of patients with PVR has been reported [54, 55]. Lee et al. demonstrated that TGF-β2 is a potent inducer of EMT in RPE cells [56]. In a model of EMT induction in the ARPE-19 cell line with TGF-β2, ZO-1 and α-SMA were downregulated and upregulated, respectively [57]. Additional data indicated that TGF-β2 activates other growth factors that contribute to PVR development. In terms of EMT signaling, Smad2/3 is the key signaling pathway of EMT induced by TGF-β, and inhibition of TGF-β via Smad2/3 signaling suppressed PVR in an experimental PVR model by knocking down Smad2/3. Blocking the Smad2/3 signaling also inhibits the expression of α- SMA, the EMT marker, and collagen IV in RPE cells. These results suggest that Smad2/3 signaling is required in the process of TGF-β- induced EMT [58]. Another study showed that TGF-β signaling induces EMT through Smad 2/3 phosphorylation, followed by Smad4 recruitment [59]. Subsequently, the complex translocates to the nucleus and interacts with certain transcription factors, which may contribute to EMT. Smad4 is an important part of the classical TGF-β pathway and plays a dual role in EMT. Zeng et al. showed that Smad4 promotes the migration, invasion and EMT of A549 cells (adenocarcinoma human alveolar basal epithelial cells) [60]. In contrast, other studies have indicated that Smad4 inhibits cancer progression [61, 62]. Authors of one study hypothesized that after Smad4 was silenced in human pancreatic tumor cells, Smad4 was still needed for TGF-β-induced cell cycle arrest and migration; however, Smad4 was not involved in TGF-β-induced EMT [63]. Meanwhile, TGF-β signaling and Smad are shown to be involved in cell cycle regulation by mediating mTOR signaling [64], while it has been well documented that mTOR plays a significant role in metabolic checkpoint regulation [65]. Consequently, further investigation is needed to determine whether miR-1285 plays any role in metabolic checkpoints via mTOR signaling.

Hidaka et al. suggested that oncogenic transglutaminase 2 (TGM2) is directly regulated by miR-1285. As in the silencing of miR-1285 after miR-1285 transfection, the silencing of target genes significantly inhibits the proliferation, invasion and migration of renal cell carcinoma (RCC) cells. The silencing modulates molecular targets in RCC cells by genes with the putative target site of miR-1285 in their 3’-UTR [66]. Previous study has reported that miR‑205 regulates the expression of Smad4 and impairs its functions in cells, therefore miR-205 is crucial for TGF-β-induced EMT, invasion and migration in NSCLC [60]. The present study expanded these findings and showed further that Smad4 downregulation also reversed cell migration and TGF-β2-induced EMT. Moreover, Smad4 is the target of miRNA-1285, which was downregulated in TGF-β-treated ARPE19 cells. In addition, miR-1285 overexpression inhibited TGF-β2-induced cell migration and EMT through Smad4 inhibition.

It has been shown that TGF-β receptor 1 inhibitor can be used for prevention of proliferative vitreoretinopathy [67]. Since TGF-β is involved in mTOR signaling and mTORC1 inhibitor-rapamycin plays a significant role in anti-proliferative properties, it will be interesting to see if rapamycin can be used for treatment purposes in vitreoretinopathy [68]. Also, the present study showed that miR-1285 overexpression can prevent TGF-β/Smad4-enhanced EMT and PVR. In a recent study, TGF-β signaling and Smad were involved in cell cycle regulation by mediating mTOR signaling [64]. Thus, we speculate that miR-1285-reduced PVR may be achieved by regulating mTOR signaling, suggesting that miR-1285 may serve as a promising therapeutic target for PVR.

In conclusion, the present study examined the regulatory effects of miR-1285 on TGF-β2-induced EMT in RPE cells, finding these effects to be mediated through downregulation of Smad4. These findings indicate that miR-1285 may constitute a new therapeutic target for PVR. It is well established that miRNAs control the cellular expression machinery via a single miRNA/multiple targets or multiple miRNAs/single target mechanism. More functional targets for miR-1285 need to be identified. Comprehensive and extensive studies of the biology of miR-1285 will shed new light on its potential roles and the pathology of retinal diseases.

## Supporting information

S1 Fig Endogenous microRNA expression amount in ARPE19 cells. (TIF)

S1 Table The criterion for each stage in the Fastenberg classification. (DOCX)

S2 Table Summary of the stage of PVR formation. (DOCX)

S1 Raw data (PDF)
