# Supplementary figures and images for: Repression of Smad4 by MicroRNA-1285 moderates TGF-β-induced epithelial–mesenchymal transition in proliferative vitreoretinopathy
Source: PLoS One. 2021 Aug 12;16(8):e0254873. doi: 10.1371/journal.pone.0254873 (PMC8360606; doi:10.1371/journal.pone.0254873)

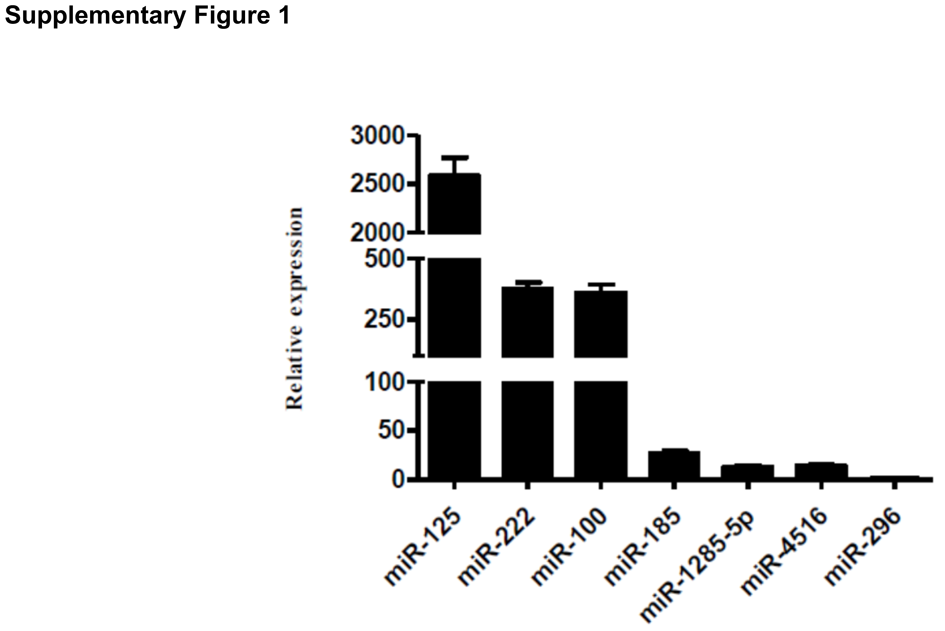

Supplement: S1 Fig — (TIF) [file pone.0254873.s001.tif]

### Figure 3A

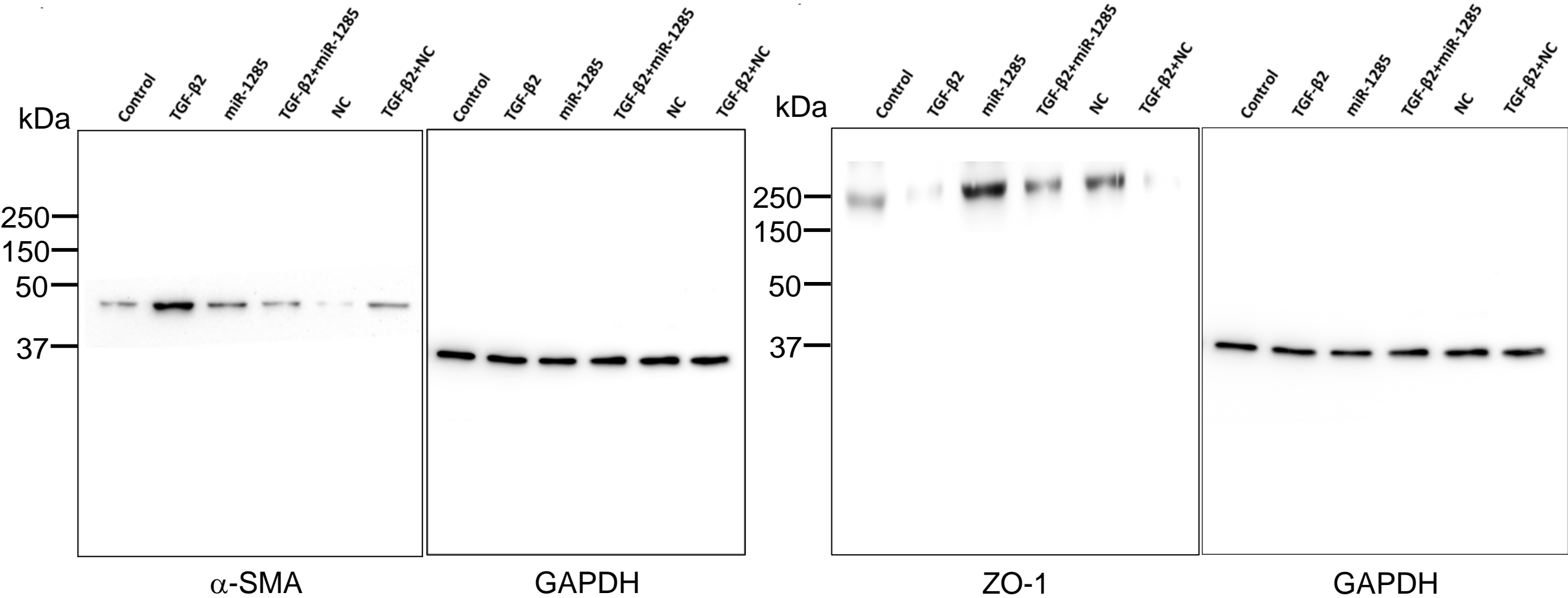

Figure 3D

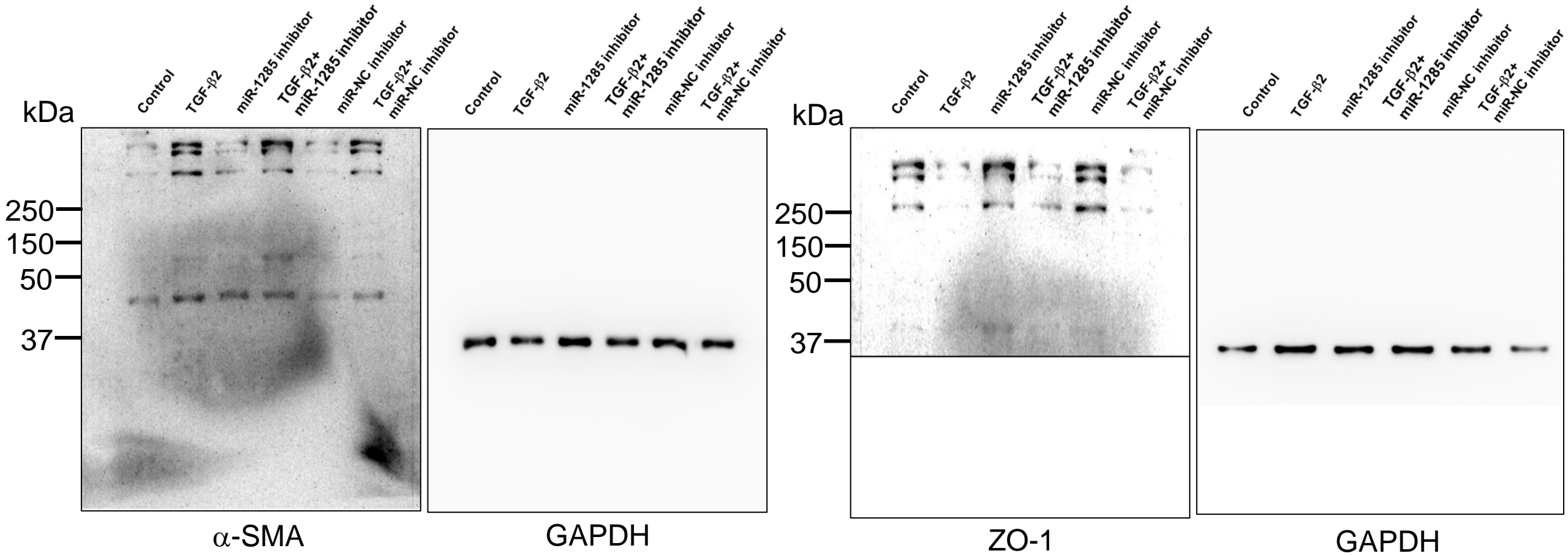

Figure 5A

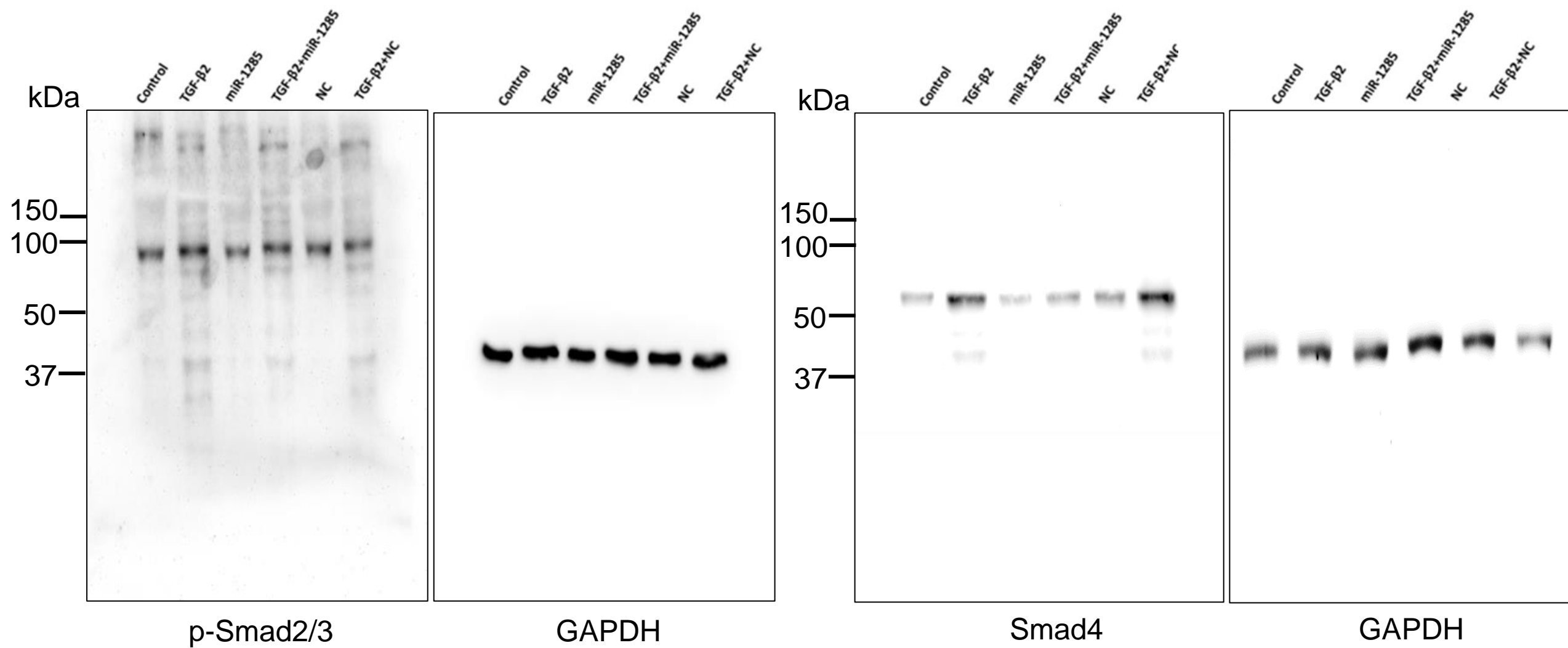

Supplement: S1 Raw data — (PDF) [file pone.0254873.s004.pdf]
