# Supplementary material for: Repression of Smad4 by MicroRNA-1285 moderates TGF-β-induced epithelial–mesenchymal transition in proliferative vitreoretinopathy
Source: PLoS One. 2021 Aug 12;16(8):e0254873. doi: 10.1371/journal.pone.0254873 (PMC8360606; doi:10.1371/journal.pone.0254873)
Supplement: S1 Table — (DOCX) [file pone.0254873.s002.docx]

S1 Table. The criterion for each stage in the Fastenberg classification.

| Classification | Description |
| --- | --- |
| Stage 0 | Normal eye |
| Stage 1 | Intravitreal membrane |
| Stage 2 | Focal traction; localized vascular changes; hyperemia; engorgement; dilation; blood vessel elevation |
| Stage 3 | Localized detachment of medullary ray |
| Stage 4 | Extensive retinal detachment; total medullary ray detachment; peripapillary retinal detachment |
| Stage 5 | Total retinal detachment; retinal folds and holes |
