# Supplementary material for: Repression of Smad4 by MicroRNA-1285 moderates TGF-β-induced epithelial–mesenchymal transition in proliferative vitreoretinopathy
Source: PLoS One. 2021 Aug 12;16(8):e0254873. doi: 10.1371/journal.pone.0254873 (PMC8360606; doi:10.1371/journal.pone.0254873)
Supplement: S2 Table — (DOCX) [file pone.0254873.s003.docx]

| **Treatment** | | **PVR stages**  **according to Fastenburg classification** | | | | |
| --- | --- | --- | --- | --- | --- | --- |
|  |  | **1** | **2** | **3** | **4** | **5** |
| Day 18 | ARPE-19 cells with miR-1285-5p | 5 | 0 | 0 | 0 | 0 |
|  | ARPE-19 cells with miR-NC | 0 | 3 | 2 | 0 | 0 |
| Day 22 | ARPE-19 cells with miR-1285-5p | 5 | 0 | 0 | 0 | 0 |
|  | ARPE-19 cells with miR-NC | 0 | 1 | 2 | 2 | 0 |
| Day 25 | ARPE-19 cells with miR-1285-5p | 5 | 0 | 0 | 0 | 0 |
|  | ARPE-19 cells with miR-NC | 0 | 0 | 1 | 2 | 2 |
| Day 29 | ARPE-19 cells with miR-1285-5p | 5 | 0 | 0 | 0 | 0 |
|  | ARPE-19 cells with miR-NC | 0 | 0 | 0 | 1 | 4 |

S2 Table. Summary of the stage of PVR formation
